# Comment on: Transmission of COVID-19 and its Determinants Among Close Contacts of COVID-19 Patients

**DOI:** 10.34172/jrhs.2022.95

**Published:** 2022-10-19

**Authors:** Zohreh Jadali

**Affiliations:** Department of Immunology, School of Public Health, Tehran University of Medical Sciences. Tehran, Iran

## Dear Editor,

 We read the article by Jashaninejad et al on determinant factors of COVID-19 transmission among close contacts of COVID-19 patients.^1^ This study demonstrates that the risk of household transmission is higher in older adults; nonetheless, no mention is made of care home staff and residents who are at higher risk of COVID-19 severe outcomes. The increased risk of acquiring COVID-19 and developing a severe disease in older adults is an issue of vital importance.^2^ There are a number of risk factors that can increase their risk of infection, including immune system ageing, movement poverty, the higher prevalence of comorbid health conditions, as well as nutrient deficiency and its related problems.^3^ Moreover, care homes are setting where older people usually live in shared accommodation; therefore, effective infection prevention and control is difficult. Other important reasons are the limited availability of medical technology or personal protective equipment and restricted staff resources. These challenges are amplified in the charities that help the elderly population and are entirely run by volunteers with no employees.

 Factors, including a limited budget, the lack of specialized nursing staff, and voluntary job abandonment, have been linked to the development and poor management of COVID-19 in such places.^4^ Infected staff also represent one of the major routes of virus transmission, and SARS-CoV-2 positivity is significantly higher among them.^5^ According to the aforementioned reports, transmission-based precautions are essential for fighting COVID-19. In this regard, health care leaders have taken various measures to control disease spread. These strategic decisions are necessary to overcome the COVID-19 challenges since several studies have found a link between excellent leadership styles and COVID-19 management.^6^

 Some of the key approaches which have been shown to reduce the risk of disease are (1) good personal hygiene (washing hands, wearing masks, keeping distances), 2)vaccine prioritization strategies targeting older people (aged ≥ 60 years), (3) regular testing for coronavirus (COVID-19) that is so important for both early diagnosis and treatment of patients, (4) separating the infected patient from other residents, (5) visitor restrictions, (6) collaboration with public health organizations and hospitals in order to increase the diagnostic tests for COVID-19, education of staff, and collaborative management.

 Although these measures have had positive impacts on COVID-19 mortality and disease transmission, there is no consensus on this issue^7^. These discrepancies can be ascribed to different reasons, including characteristics of disease [asymptomatic vs symptomatic transmission), characteristics of residents (comorbidities, nutritional status, physical and cognitive factors), facility characteristics (space allocation and occupancy), staffing-related factors (ratios of staff to residents, inadequate staffing), and other factors, such as different circulation pattern of virus in different geographical areas or poverty.

 The past international experience of COVID-19 reflects the benefits of disease prevention programs. Nonetheless, the increasing rate of COVID-19 cases and deaths in residential care homes for the elderly indicates a gap in knowledge and system weakness. The removal of barriers needs successful planning on the basis of information analysis and strict monitoring for measuring progress towards the agreed objectives. Effective leadership and intersectional collaboration are the keys to success in disease control and mitigation.

 Overall, the pandemic has presented multiple challenges for care homes, and various regulations have been proposed to reduce the burden of disease on both residents and staff. At present, there is a dearth of information on the benefits and drawbacks of COVID-19-related preventive behaviors. It seems that focusing on minimizing or eliminating COVID-19 is a necessary but not a sufficient condition for disease control and prevention. Guidelines for maximizing the health of residents and staff also appear to have discernible benefits for future pandemic preparedness.
