# Supplementary material for: Comment on: Transmission of COVID-19 and its Determinants Among Close Contacts of COVID-19 Patients
Source: J Res Health Sci. 2022 Oct 19;22(3):e00560. doi: 10.34172/jrhs.2022.95 (PMC10422151; doi:10.34172/jrhs.2022.95)
Supplement: Related texts

# Reply: Comment on: Transmission of COVID-19 and its Determinants Among Close Contacts of COVID-19 Patients

**Published:** 2022-10-19

**Authors:** Manoochehr Karami

**Affiliations:** Department of Epidemiology, School of Public Health & Safety, Shahid Beheshti University of Medical Sciences, Tehran, Iran

## Authors' Reply

### Dear Editor,

Thank you for showing interest in our published article^1^. Sharing all of the recommendations and highlights of the lesson learned from the COVID-19 epidemic by the author(s) regarding the prevention and transmission of COVID-19 is excellent. The author (s) have addressed the aspects of COVID-19-related outcomes in long-term care and facilities. They pointed out our findings in the case of a higher secondary attack rate of COVID-19 infection among the elderly contacts in comparison with other household members and close contacts of index cases. Nonetheless, the author(s) did not make criticism over findings and focused on justification and clarification of determinates of their own interesting results of our article. However, we would like to inform the kind author(s) and journal’s readership that we aimed to describe the distribution of transmission risk among close contacts to provide public health authorities with substantial evidence.

## References

[1] Jashaninejad R, Doosti-Irani A, Karami M, Keramat F, Mirzaei M (2021 Apr 25). Transmission of COVID-19 and its Determinants among Close Contacts of COVID-19 Patients Running title. J Res Health Sci.
